# Colonization rate of *Streptococcus pneumoniae*, its associated factors and antimicrobial susceptibility pattern among children attending kindergarten school in Hawassa, southern Ethiopia

**DOI:** 10.1186/s13104-019-4376-z

**Published:** 2019-06-17

**Authors:** Aberash Assefa Haile, Deresse Daka Gidebo, Musa Mohammed Ali

**Affiliations:** Hawassa University College of Medicine and Health Sciences School of Medical Laboratory Science, P.O box 1560, Hawassa, Ethiopia

**Keywords:** *S. pneumoniae*, Nasopharyngeal colonization, Hawassa

## Abstract

**Objective:**

The aim of this study was to determine the colonization rate of *Streptococcus pneumoniae*, antimicrobial susceptibility pattern and associated risk factors among children attending kindergarten school in Hawassa, Ethiopia.

**Results:**

Out of 317 study participants, 68 (21.5%) were colonized with *S. pneumoniae*. Colonization rate was significantly associated with factors such as age (3 to 4 years old) (P = 0.01), having a sibling whose age was less than 5 years (P = 0.011), sharing a bed with parents (P = 0.005), cooking within bedroom (P = 0.002), and previous hospitalization (P = 0.004). Forty-four (64.6%), 33 (48.5%), and 2942.6%) of *S. pneumoniae* isolated were resistant to cotrimoxazole, penicillin, and tetracycline respectively.

## Introduction

*Streptococcus pneumoniae* (*S. pneumoniae*) is the major cause of childhood morbidity and mortality in the world [1, 2]. In Africa, *S. pneumoniae* caused 1–4 million episodes of disease among under-five children in 2007 [3]. In Ethiopia, 21.4% of invasive disease among children and adults were caused by *S. pneumoniae* [4, 5]. Even though pneumococcal conjugate vaccine (PCV-10) was introduced in Ethiopia since 2011 as part of childhood immunization, 43.3% of meningitis and sepsis were caused by *S. pneumoniae* among children in 2015 [6].

Nasopharyngeal colonization of *S. pneumoniae* always precedes disease and serves as a reservoir for the transmission of the pathogen within the community [1]. The colonization rate varies depending on age, vaccination status, and other factors [7]. The rate of *S. pneumoniae* colonization is particularly high among young children attending kindergarten schools [8, 9]. Vaccination status is assumed to reduce the colonization rate among children; however, its effect on colonization was not assessed in most low-income countries including Ethiopia. Factors such as environmental [10], socio-demographic [11] and previous health condition [8] can increase the prevalence of *S. pneumoniae* among school children. Moreover, nasopharyngeal colonization with antibiotic-resistant *S. pneumoniae* has been increasing in different parts of the world [10, 12, 13]. The rise of colonization rate with antibiotic-resistant *S. pneumoniae* creates a challenge to treat the disease caused by *S. pneumoniae* among carriers [10, 14].

Most of the studies focusing on *S. pneumoniae* in Ethiopia were conducted among sick children [4, 5]. Data on the *S. pneumoniae* colonization rate, associated risk factors, and antimicrobial susceptibility pattern among kindergarten school children were scarce in the study area. Therefore the present study was aimed to determine colonization rate, associated factors and antimicrobial susceptibility pattern of circulating *S. pneumoniae* among kindergarten school children in Hawassa, the southern part of Ethiopia.

## Main text

### Methods

#### Study area and period

Hawassa is the capital city of the Southern Nations, Nationalities and People Regional State. The city is located on the shores of Lake Hawassa in the Great Rift Valley and is located 275 km to the south of Addis Ababa, the capital of Ethiopia [15].

#### Study design

A community-based cross-sectional study was conducted from March 17, 2018, to May 20, 2018, at Tabor sub city, Hawassa, Ethiopia.

#### Study population

All children who attended kindergarten schools found in Tabor sub city, Hawassa, Ethiopia during the study period.

#### Sample size determination

Sample size was calculated by using a single proportion formula.

$$ n = \frac{{Z^{2} P\left( {1 - P} \right)}}{{d^{2} }} $$

where n: sample size; Z: reliability coefficient (confidence level) which is 95% = 1.96; P: anticipated population proportion; D: the margin of error, which is 5% = 0.05.

By using the anticipated population proportion of 44.8% from a previous study [8], the sample size was calculated as follows:

$$ n = \frac{{\left( {1.96} \right)^{2} 0.448\left( {1 - 0.448} \right)}}{{\left( {0.05} \right)^{2} }} = 380 $$

Since the source population was less than 10,000, the sample size was recalculated using the correction factor as follows:

$$ {\text{nf }} = \frac{n}{{1 + \frac{n}{N}}} \to \frac{380}{{1 + \frac{380}{1302}}} = 294.6. $$

After adding 10% of non-respondent the final the sample size was 324.

#### Sampling technique

At Tabor sub city, there are seven primary schools with a total of 1302 children attending kindergarten. Out of the total children, 324 were selected randomly with an equal proportion from each school. From 324 selected children, seven refused to participate in the study.

#### Inclusion criteria

Children who attended kindergarten school during the study period, those whose age was less than or equal to 6 years, and children whose parents had accepted the consent to participate in the study were included.

#### Exclusion criteria

Children who were on an antibiotic for the last 3 weeks, those with any sign and symptoms of respiratory disease and those who were not brought to school by their parents or guardians were excluded from the study.

#### Data collection

At each selected school, the parents of the children were consented and interviewed for sociodemographic, the environmental and previous health condition of the children by the trained data collectors. Data was collected by using pre-structured and pre-tested questionnaire.

#### Sample collection, handling, and transport

The nasopharyngeal specimen was collected by passing sterile rayon-tipped swab gently back from one nostril along the floor of the nasal cavity until it touches the posterior wall of the nasopharynx. One nasopharyngeal specimen per child was collected by a trained nurse.

#### Culture and identification of *S. pneumoniae*

The specimens were inoculated onto sheep blood agar (Oxoid Ltd, CM0271) supplemented with 5 μg/ml gentamycin by rolling the swab over a small area of the plate and stretching the sample using a sterile loop within 3 h of collection. The inoculated media was incubated in 5% CO_2_ enriched atmosphere at 37 °C for 18 to 20 h. Suspected colonies, which appears as a greenish colony (alpha hemolytic), were subcultured on blood agar into which 5 μg Optochin disks (Mumbai, India) was placed, and then incubated within 5% CO_2_ enriched atmosphere at 37 °C for 24 h. After overnight incubation, the sensitivity of Optochin was checked by measuring the diameter of the inhibition zone. If the diameter was ≥ 14, it was assumed to be *S. pneumoniae*; if the diameter was < 14, bile solubility tests (tube method) was performed by using 2% sodium deoxycholate (Oxoid Ltd) [16].

#### Antimicrobial susceptibility testing

Antimicrobial susceptibility pattern of all *S. pneumoniae* isolated was assessed by using the disk diffusion method. The antibiotics used were tetracycline (30 μg), cotrimoxazole (23.75 μg), oxacillin (1 μg), and chloramphenicol (30 μg), erythromycin (15 μg), clindamycin (2 μg), and rifampicin (5 μg).

#### Data management and quality control

Quality of the data was ensured by using a pre-structured questionnaire. For laboratory analysis, the sterility of the prepared media was checked by incubating 5% of prepared media within a 5% CO_2_ enriched atmosphere at 37 °C for 24 h before using it. A quality control strain of *S. pneumoniae* was used as a positive control for each test.

#### Data processing and analysis

Data were analyzed by using SPSS version 22. The frequency of variables, the prevalence of *S. pneumoniae,* and antibiotic susceptibility pattern was determined by using SPSS version 22. The association between risk factors and *S. pneumoniae* colonization was determined by using logistic regression. A P-value less than 0.05 at 95% confidence interval (CI) was considered statistically significant.

### Results

#### Sociodemographic and health characteristics

Out of 317 children who participated in the present study, 154 (48.6%) were males, 262 **(**82.6%**)** were within the age of 5 to 6 years, 17 (5.4%) were not vaccinated, and 13 (4.1%) lived with smoker families (Table 1).

**Table 1:** Sociodemographic and health characteristics of children who attended kindergarten school owned by government at Tabor sub city, Hawassa from March 17, 2018 to May 20, 2018, (N = 317)

Characteristics	N (%)
Age	
3–4	55 (17.4)
5–6	262 (82.6)
Sex	
Male	154 (48.6)
Female	163 (51.4)
Sibling < 5 years old	
Yes	216 (68.1)
No	101 (31.9)
Number of sibling per house	
1	43 (19.9)
≥ 2	173 (80.1)
Daycare attendance	
Yes	104 (32.8)
No	213 (67.2)
Smoker in a house	
Yes	13 (4.1)
No	304 (95.9)
Sharing bed with parents	
Yes	213 (67.2)
No	104 (32.8)
Cooking in bed room	
Yes	168 (53.0)
No	149 (47.0)
Number room per house	
1	218 (68.8)
≥ 2	99 (31.2)
Number of family in the house	
< 5	210 (66.2)
≥ 5	107 (33.8)
Previous disease	
Yes	218 (68.8)
No	99 (31.2)
Previous hospitalization	
Yes	39 (17.9)
No	179 (82.1)
Vaccination status	
Yes	300 (94.6)
No	17 (5.4)

N: total participated children; n: number; %: percentage

#### Nasopharyngeal colonization rate of *S. pneumoniae*

The colonization rate of *S. pneumoniae* among children who attended kindergarten was 68 (21.5%) 95% CI [17–26.2]. Colonization rate of *S. pneumoniae* among children who were 3 to 4 years old, 5 to 6 years old, those who were not vaccinated, and those who live with a smoker were 19 (34.5%), 49 (18.7%), 5 (29.4%), and 5 (38.5%) respectively (Table 2).

**Table 2:** Factors that affect nasopharyngeal colonization rate of *S. pneumoniae* among children who attended kindergarten school owned by government at Tabor sub city, Hawassa from March 17, 2018 to May 20, 2018, (N = 317)

Variables	Colonization rate		COR (95% CI)	P value	AOR (95% CI)	P value
	No (%)	Yes (%)				
Age						
3–4	36 (65.5)	19 (34.5)	2.3 (1.2–4.3)	0.011	3.1 (1.3–7.6)	0.010
5–6	213 (81.3)	49 (18.7)	1		1	
Sex						
Male	118 (76.6)	36 (23.4)	1.2 (0.7–2.1)	0.417	–	–
Female	131 (80.4)	32 (19.6)	1			
Sibling < 5 years old						
Yes	160 (74.1)	56 (25.9)	2.6 (1.3–5.1)	0.006	2.9 (1.3–6.8)	0.011
No	89 (88.1)	12 (11.9)	1		1	
Number of sibling per house						
1	29 (67.4)	14 (32.6)	1.5 (0.7–3.1)	0.269	–	–
≥ 2	131 (75.7)	42 (24.3)	1			
Daycare attendance						
Yes	86 (82.7)	1 8 (17.3)	0.7 (0.4–1.2)	0.211	0.7 (0.3–1.5)	0.345
No	163 (76.5)	50 (23.5)	1		1	
Smoker in a house						
Yes	8 (61.5)	5 (38.5)	2.4 (0.8–7.6)	0.138	2.6 (0.6–12.3)	0.225
No	241 (79.3)	63 (20.7)	1		1	
Sharing bed with parents						
Yes	157 (73.0)	58 (27.0)	2.7 (1.4–5.4)	0.003	3.5 (1.4–8.4)	0.005
No	92 (90.2)	10 (9.8)	1		1	
Cooking in bed room						
Yes	118 (70.2)	50 (29.8)	3.1 (1.7–5.6)	< 0.001	3.6 (1.6–7.9)	0.002
No	131 (87.9)	18 (12.1)	1		1	
Number room per house						
1	169 (77.5)	49 (22.5)	1.2 (0.7–2.2)	0.509	–	–
≥ 2	80 (80.8)	19 (19.2)	1			
Number of family in the house						
< 5	166 (79.1)	44 (20.9)	0.9 (0.5–1.6)	0.762	–	–
≥ 5	83 (77.6)	24 (22.4)	1			
Previous disease						
Yes	168 (77.1)	50 (22.9)	1.3 (0.7–2.4)	0.340	–	–
No	81 (81.8)	18 (18.2)	1			
Previous hospitalization						
Yes	21 (53.8)	18 (46.2)	3.9 (1.9–8.2)	< 0.001	3.5 (1.5–8.2)	0.004
No	147 (82.1)	32 (17.9)	1		1	
Vaccination status						
Yes	237 (79.0)	63 (21.0)	0.6 (0.2–1.9)	0.415	–	–
No	12 (70.6)	5 (29.4)	1			

N: total participated children; COR: crud odds ratio; AOR: adjusted odds ratio, CI: confidence interval, %: percentage

#### Factors that affect the nasopharyngeal colonization rate of *S. pneumoniae*

In this study variables which are listed below were significantly associated with colonization rate of *S. pneumoniae* among children who attended kindergarten: children within the age range of 3 to 4 years (Adjusted odds ratio (AOR) = 3.1; 95% CI [1.3–7.6]; P = 0.01), having sibling with age < 5 years old (AOR = 2.9; 95% [CI 1.3–6.8]; P = 0.011), sharing bed with parents (AOR = 3.5;95% CI [1.4–8.4]; P = 0.005), cooking within bedroom (AOR = 3.6; 95% CI [1.6–7.9]; P = 0.002) and previous hospitalization (AOR = 3.5; 95% CI [1.5–8.2]; P = 0.004) (Table 2).

Out of 68 of *S. pneumoniae* isolated in this study, 67 (98.5%), 65 (95.6%), 62 (91.2%), and 58 (85.3%) were susceptible to rifampicin, clindamycin, chloramphenicol, and erythromycin respectively. Of all isolates, only three were sensitive to all of the seven antibiotics tested, 17 (25%) were resistant to only one antibiotic, 12 (17.6%) were resistant to two antibiotics and 2 (2.9%) were resistant for three antibiotics. Multidrug-resistant (resistant for three and more antibiotics) from this finding was 2 (2.9%). Majority of *S. pneumoniae* isolated in this study were resistant to cotrimoxazole 44 (64.6%), oxacillin 33 (48.5%) and tetracycline 29 (42.6%) (Table 3).

**Table 3:** Antimicrobial susceptibility pattern of *S. pneumoniae* isolated from children who attended kindergarten school owned by government at Tabor sub city, Hawassa from March 17, 2018 to May 20, 2018, (N = 68)

Antimicrobial agents	Resistant	Susceptible	Intermediate
	n (%)	n (%)	n (%)
Tetracycline	29 (42.6)	23 (33.8)	16 (23.6)
Erythromycin	3 (4.4)	58 (85.3)	7 (10.3)
Clindamycin	3 (4.4)	65 (95.6)	0 (0)
Rifampicin	0 (0)	67 (98.5)	1 (1.5)
Cotrimoxazole	44 (64.6)	12 (17.7)	12 (17.7)
Chloramphenicol	6 (8.8)	62 (91.2)	–
Oxacillin	33 (48.5)	35 (51.5)	–

N: isolated organism; n: number; %: percentage

### Discussion

The *S. pneumoniae* colonization rate among children who attended kindergarten school in the present study was 21.5%. The finding of this study was higher than the study conducted in the northern part of Ethiopia (10.3%) [17], Gambia (7.6%) [18] and Tanzania (12.3%) [19]. *S. pneumoniae* colonization rate found in this study was low compared to report from Gondar, Ethiopia (41%) [10], Jimma, Ethiopia (43%) [14] and Kenya (35%) [20]. The possible explanation for the variation might be due to vaccination status and age differences. This indicates that children whose age is below 6 years old are more colonized compared with those who are above 6 years old. Additional explanations for the differences observed are sample size, seasonal variation and method used. Even though the nasopharyngeal colonization rate of *S. pneumoniae* among children varies throughout the world, the result of this study was in line with the colonization rate reported from Nigeria [21], Morocco [22] and Kenya [23].

In the present study, we assessed different factors that could possibly increase the colonization rate of *S. pneumoniae*. Children whose age was in between 3 and 4 years were 3.1 times at risk to be colonized with *S. pneumoniae* (P = 0.01). This finding was in line with studies conducted in Belgium, Spain, and Ethiopia [10, 24, 25]. The decline in *S. pneumoniae* colonization rate as age increases could be due to the gradual acquisition of mucosal immunity and reduction of exposure. This indicates children whose age was in between 3 and 4 year were at high risk of acquiring *S. pneumoniae* colonization than those whose age was in between 5 and 6 years. Children who lived together with a sibling(s) whose age was less than 5 years old had 2.9 times chance to be colonized with *S. pneumoniae* compared to children who did not have a sibling whose age was not less than 5 years old (P = 0.001). This finding was comparable with a report from other parts of Ethiopia [10, 14].

Unlike previous studies from Ethiopia [10, 14], children who did not have their own separate bed in the present study had 3.5 times chance to be colonized with *S. pneumoniae* (P = 0.005). Children who sleep in the cooking room had 3.6 times chance to be colonized with *S. pneumoniae* compared with children who sleep in a bedroom which was free from cooking (P = 0.002). This finding was comparable with a report from Kenya [26]. Children with a history of hospital admission had 3.5 times chance to be colonized with *S. pneumoniae* (P = 0.004). This finding was consistent with a study conducted in France and Uganda [27, 28].

Factors such as attending daycare centers and being passive smoker were not significantly associated with *S. pneumoniae* colonization during multivariable analysis. In contrast to the present study, a significant association of daycare attendance and passive smoking with *S. pneumoniae* colonization rate was reported from other countries [14, 29, 30]. In the current study, a high proportion (21%) of vaccinated children were colonized with *S. pneumoniae* even though it was not statistically significant (P < 0.05).

Out of the total *S. Pneumoniae* strains collected in the present study, 64.6% and 42.6% were resistant to cotrimoxazole and tetracycline respectively. This finding was comparable with previous studies conducted in different parts of Ethiopia [8, 10, 14] and Morocco [31]. The prevalence of tetracycline resistant *S. pneumoniae* reported from Iran (22.6%) was low compared to our study [32]. Unlike the current study, the majority of *S. pneumoniae* isolated from Kenya (98.6%) were resistant to cotrimoxazole [33]. Wide usage of antibiotics could be one of the reasons for high resistance rate observed in different countries. The prevalence of penicillin (oxacillin) resistant *S. pneumonia*e we found (48.5%) was low compared to the report Kenya (82%) [33].

According to our observation, there is wide use of cotrimoxazole in the study area for the treatment of pneumonia. Moreover, most of *S. pneumoniae* isolates collected in this study were resistant to cotrimoxazole indicating the importance of revising the treatment guideline for pneumonia.

## Limitations

Penicillin resistance was performed by using a modified disk diffusion method. Not able to type *S. pneumoniae* isolates.

## Data Availability

The data used/analyzed during the current study available from the corresponding author on reasonable request.

## Abbreviations

*S. pneumoniae*: *Streptococcus pneumoniae*
PCV: pneumococcal conjugate vaccine
CI: confidence interval
AOR: adjusted odds ratio
